# EDX-analysis of fluoride precipitation on human enamel

**DOI:** 10.1038/s41598-019-49742-5

**Published:** 2019-09-17

**Authors:** Konstantin Johannes Scholz, Marianne Federlin, Karl-Anton Hiller, Helga Ebensberger, Gerlinde Ferstl, Wolfgang Buchalla

**Affiliations:** Department of Conservative Dentistry and Periodontology, University Medical Center Regensburg, Regensburg, Germany

**Keywords:** Electron microscopy, Preclinical research

## Abstract

One mechanism of action for the anticaries effect of topical fluoridation is through precipitation of CaF_2_. In this *in vitro* study energy-dispersive x-ray spectroscopy (EDX) is used as a semiquantitative method to detect enamel fluoride-precipitation under the influence of acidic and neutral pH-value and absence or presence of a salivary pellicle. Crowns of 30 human caries-free third molars were quartered into four specimens and the enamel surface ground flat and polished. Two specimens each were stored in human saliva (120 minutes pellicle formation). Teeth were randomly allocated into 6 treatment groups: NaF_a (experimental acidic sodium fluoride; 12500 ppmF^−^, pH 4.75); NaF_n (experimental neutral sodium fluoride; 12500 ppmF^−^, pH 7.0); GB_a (acidic gel base; 0 ppmF^−^, pH 4.75); GB_n (neutral gel base; 0 ppmF^−^, pH 7.0); AmF-NaF_a (experimental acidic amine/sodium fluoride; 12500 ppmF^−^, pH 4.75); EG_a (acidic amine/sodium fluoride; Elmex Geleé, CP-GABA GmbH; 12500 ppmF^−^, pH 4.75). Each gel was applied for 60 seconds to one specimen with and one specimen without pellicle. Two specimens served as controls (no gel, without/with pellicle). Atomic percent (At%) of O, F, Na, Mg, P, Ca was measured by EDX. ∆At% and Ca/P-ratios were calculated. EDX could semi-quantify superficial enamel fluoride-precipitation. Only specimens treated with acidic fluoride gels showed fluoride-precipitation, a salivary pellicle tended to decrease At%F.

## Introduction

For over 75 years studies confirm the caries-preventive effect of water fluoridation above 1 parts per million (ppm) on caries prevelance and composition of teeth^1–5^. The benefits of fluoride toothpastes with concentrations above 1,000 ppmF^−^ for caries prevention are also widely accepted^6^. Fluorine contents of foods and teeth in early reports are based on estimations using microtitration with thorium nitrate or comparative colorimetry after ashing the samples and isolating the fluorine by steam distillation^7^. At that time, systemical fluoridation during odontogenesis has been presumed to be the most important pathway of caries prevention through fluorides^8^. Nowadays, fluorides are considered to work as a key agent in preventing caries primarily through topical mechanisms^9–11^. Deposition of CaF_2_-like complexes on enamel surfaces following topical fluoridation by high fluoride containing agents with low pH-value has been frequently described^12^. So far, it is not exactly known if it is pure CaF_2_ or if there were other components in these complexes^13,14^. It is likely that those complexes serve as a pool for release of fluoride ions, which enable an inhibition of demineralization by adsorption on partially demineralized crystals^15^. The remineralization of initial carious lesions requires Ca and PO_4_ from saliva, but is facilitated by presence of fluorides^16^. The presence of saliva on the one side lowers the concentration level of applied fluorides. On the other side, components of human saliva such as proteins and dibasic phosphate are believed to be a potential cofactor for precipitation by means of coating CaF_2_ and serving as reservoir for calcium ions^17^. Previous studies suggest that CaF_2_-precipitation is enhanced on a clean enamel surface without plaque or pellicle, for high fluoride concentrations, long exposure period and low pH of the fluoride solution or gel^12,18,19^.

The fluoride ion electrode is widely used for measuring fluoride ion concentrations in solution. CaF_2_-like fluoride precipitates can be dissolved by alkaline solutions before further processing for measuring with the fluoride electrode (KOH-soluble fluoride). Determination of structurally bound fluoride requires acid dissolution of enamel layers of known thickness or volume, e.g. by use of perchloric acid^20–22^. KOH-dissolution of CaF_2_-like fluoride precipitates and acid dissolution of enamel can be combined in order to separate the “loosely bound”, KOH-soluble, from the “structurally bound”, acid soluble, fluoride fraction. It has been shown that scanning electron microscopy (SEM) techniques with or without energy-dispersive x-ray spectroscopy (EDX) are suitable methods for characterizing single KOH-soluble precipitates on human dental enamel^12^, but these methods are still not widely used for characterization of the enamel surface following topical fluoride application.

## Aim of The Study

The aim of this *in vitro* study was to evaluate superficial CaF_2_-precipitation on human enamel measured in atomic percent fluorine (At%F) using EDX after treatment with different gels with or without fluoride. Further parameters to be investigated were different fluoride gel formulations, the pH-value of the gel and absence or presence of a salivary pellicle. The precipitation of CaF_2_ measured in At%F was evaluated after treating human enamel with a marketed (EG_a) and an experimental (AmF-NaF_a) acidic gel comprising sodium fluoride and amine fluoride, an acidic gel just with sodium fluoride (NaF_a) and a neutral gel with sodium fluoride (NaF_n). The other tested gels were acidic (GB_a) and neutral (GB_n) gel bases without fluoride.

## Results

### Semiquanitative EDX analysis

**Figure 1:**
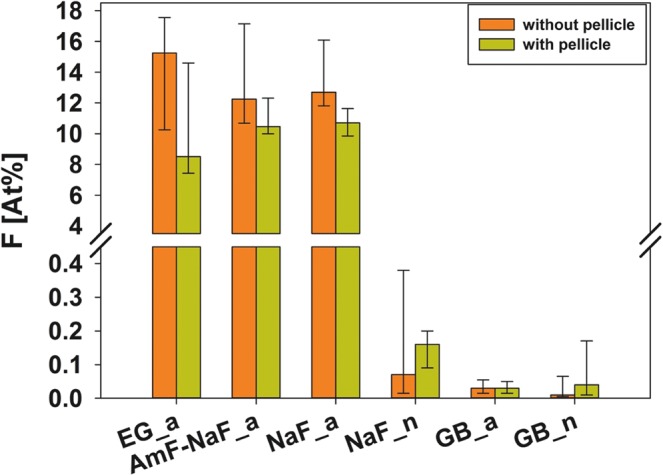
Relative atomic surface fluorine content determined by EDX. Median and 25–75% percentiles for each test group. For group allocation, see Table 3.

The At%F (atomic percent fluorine) of groups NaF_n, GB_a and GB_n was below 0.2 regardless of the presence of a pellicle. Calculation of coefficients of variability for every single specimen revealed a maximum of 13.7% without and 7.3% with pellicle for EG_a, AmF-NaF_a and NaF_a. From these groups with acidic fluoride gels At%F group-medians with pellicle ranged from 8.5 to 10.7. Without pellicle group-medians of At%F were in between 12.3 and 15.3 (Table 1, Fig. 1). In general, Error Rates Method (k = 3) revealed that the influence of pellicle was not significant. The pairwise consideration showed that the influence of pellicle was significant only for NaF_a (p = 0.016; Table 2).

**Table 1:** Semiquantitative results of atomic percent [At%] of all evaluated elements and Ca/P-ratio for test materials and controls without and with pellicle.

Element	Pellicle	Gel	EG_a		AmF-NaF_a		NaF_a		NaF_n		GB_a		GB_n	
			Median	25–75%	Median	25–75%	Median	25–75%	Median	25–75%	Median	25–75%	Median	25–75%
F	−	−	<0.1	0.0-<0.1	<0.1	<0.1–0.2	<0.1	<0.1-<0.1	<0.1	<0.1–0.7	0.1	<0.1–0.1	0.1	<0.1–0.1
	−	+	15.3	10.3–17.6	12.3	10.7–17.1	12.7	11.8–16.1	0.1	<0.1–0.4	<0.1	<0.1–0.1	<0.1	<0.1–0.1
	+	−	<0.1	0-<0.1	<0.1	<0.1–0.1	<0.1	0-<0.1	<0.1	<0.1–0.5	<0.1	<0.1–0.1	0	0-<0.1
	+	+	8.5	7.4–14.6	10.5	10.0–12.3	10.7	9.9–11.6	0.2	0.1–0.2	0.0	0.0-<0.1	0.0	0.0–0.2
O	−	−	63	59.4–67.1	62.7	61–71	62.9	59.1–64.4	60.4	58.8–69	61.4	57.3–64.6	61.4	57.6–64.3
	−	+	51.7	47–52.7	51.8	45.6–53.3	50.9	41.9–51.4	61.4	57.5–62.4	63.1	62.2–64.9	62.9	57.5–64.9
	+	−	65.1	57.8–67.8	61.5	57.7–65.5	59	55.4–61.2	64.4	61.6–65.8	59.9	59.5–64.3	60	55.8–61.5
	+	+	55.2	51.3–59.6	53	50–54.6	50.4	42.2–53.8	64.5	62.1–66.9	62.6	60.4–63.4	59.8	58.5–65.2
Na	−	−	1	1–1.2	1.1	0.8–1.1	1.1	1–1.2	1	1–1.2	1	0.9–1.1	1.1	1–1.3
	−	+	1.6	1.4–2.3	1.7	1.5–2.1	1.7	1.5–1.8	1.1	1–1.5	1	0.9–1	1	0.9–1.1
	+	−	0.9	0.8–1.1	1	1–1.2	1	1–1.1	1	1–1.6	1.1	0.9–1.2	1	1–1.2
	+	+	1.4	1.2–1.6	1.4	1.4–1.6	1.6	1.3–1.6	1	1–1.2	1	1–1	1.2	1.2–1.4
Mg	−	−	0.3	0.3–0.7	0.4	0.2–0.4	0.4	0.3–0.5	0.4	0.2–0.5	0.5	0.3–0.6	0.4	0.3–0.6
	−	+	0.4	0.3–0.7	0.4	0.4–0.6	0.4	0.4–0.5	0.4	0.4–0.6	0.3	0.3–0.4	0.4	0.3–0.5
	+	−	0.3	0.2–0.4	0.4	0.3–0.5	0.5	0.4–0.5	0.3	0.3–0.6	0.5	0.3–0.5	0.5	0.4–0.5
	+	+	0.3	0.3–0.6	0.4	0.4–0.4	0.4	0.4–0.5	0.3	0.3–0.4	0.4	0.3–0.4	0.4	0.4–0.6
P	−	−	13.8	13.1–15.1	13.8	11.8–14.4	13.8	13.2–15	14.7	12.4–15.2	14.3	13.2–15.7	14.4	13.4–15.7
	−	+	12.8	10.9–13	12.6	12.4–12.7	13	12.3–13.5	14.6	14–15.8	13.8	13.2–14.2	14.1	13.2–15.7
	+	−	13.5	12.4–15.6	14.4	13.1–15.5	15.2	14.4–16.3	13.4	13.1–14.3	14.8	13.4–15	14.9	14.3–16.3
	+	+	12	11.3–13	12.8	12.3–13	13.4	12.4–15.8	13.5	13.1–14	14	13.8–14.8	14.9	13.6–15.3
Ca	−	−	21.8	18.4–23.8	22	16.1–23.1	22	21.1–24.1	23.4	16.7–24.2	22.9	20.8–25.2	22.8	20.9–24.7
	−	+	21.5	19.5–23.3	21.7	21.4–22.3	22.3	22–26.3	22.4	21.6–25.3	21.5	20.6–22.4	21.7	20.7–25.2
	+	−	19.9	18.9–25.2	22.7	20.1–25	24.2	22.9–26.8	20.3	18.7–22.8	23.4	21.2–23.9	23.6	22.7–26.4
	+	+	20.5	17.1–22.6	22.4	20.7–23	23.7	21.5–29	20.2	18.5–22.4	22.1	21.5–23.4	23	19.5–24.4
Ca/P-ratio	−	−	1.57	1.38–1.59	1.59	1.36–1.6	1.6	1.59–1.61	1.59	1.29–1.6	1.59	1.57–1.61	1.58	1.55–1.59
	−	+	1.79	1.66–1.87	1.72	1.7–1.79	1.82	1.75–1.94	1.58	1.52–1.61	1.57	1.54–1.59	1.59	1.54–1.61
	+	−	1.59	1.46–1.61	1.59	1.53–1.61	1.6	1.59–1.64	1.52	1.42–1.59	1.59	1.57–1.6	1.59	1.59–1.62
	+	+	1.68	1.52–1.75	1.75	1.66–1.81	1.77	1.73–1.84	1.5	1.42–1.6	1.58	1.56–1.59	1.59	1.4–1.6

**Figure 2:**
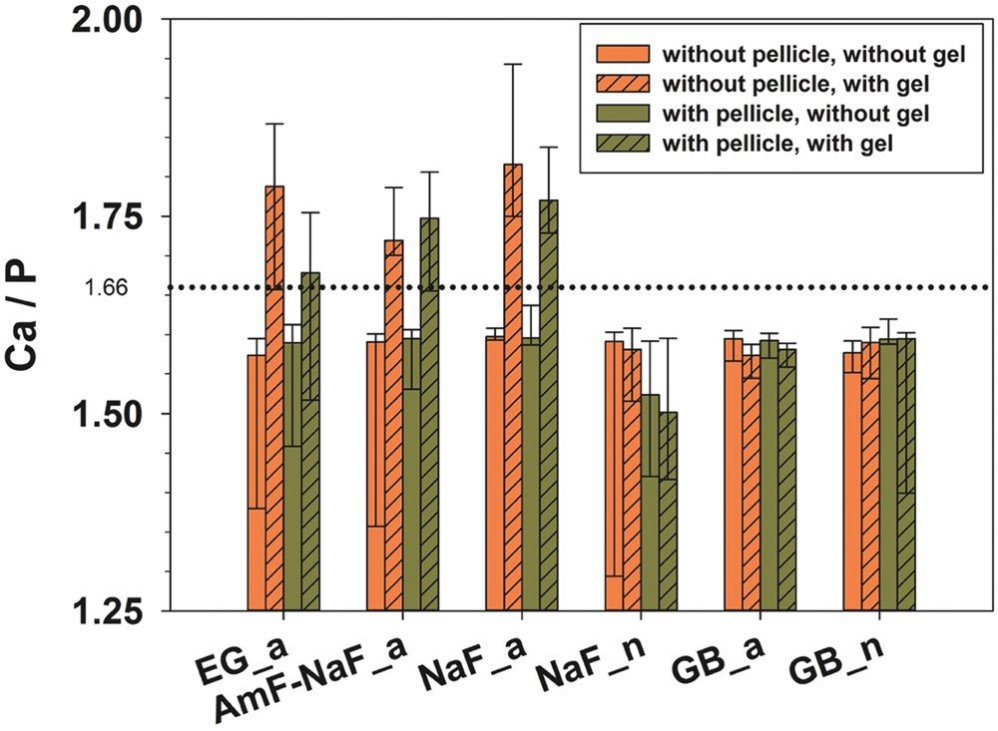
Ca/P-ratio of all specimens (Median and 25–75% percentiles); dotted line shows stoichiometric ratio (1.66) of hydroxy apatite. Data also shown in Table 1.

The value of each single tooth is the median of the 3 measurement areas in the ROI (shown in Fig. 9). Since data are non-symmetrically distributed, some of the sums of the medians may differ from 100%. The Ca/P ratio is also depicted in Fig. 2.

**Table 2:** Pairwise comparisons of fluoride-precipitation (At%F) of tested gels without and with pellicle; n.s. = statistically not different (p > 0.05).

Without pellicle	EG_a	AmF-NaF_a	NaF_a
With pellicle			
EG_a	n.s.	n.s.	n.s.
AmF-NaF_a	n.s.	n.s.	n.s.
NaF_a	n.s.	n.s.	0.032

The Ca/P-ratio was similar for all control specimens without gel. Control specimens, NaF_n, GB_a and GB_n had a Ca/P-ratio below 1.66. Specimens treated with acidic, fluoride containing gels showed Ca/P-ratio above the stoichiometric ratio of 1.66 (Fig. 2, Table 1).

**Figure 3:**
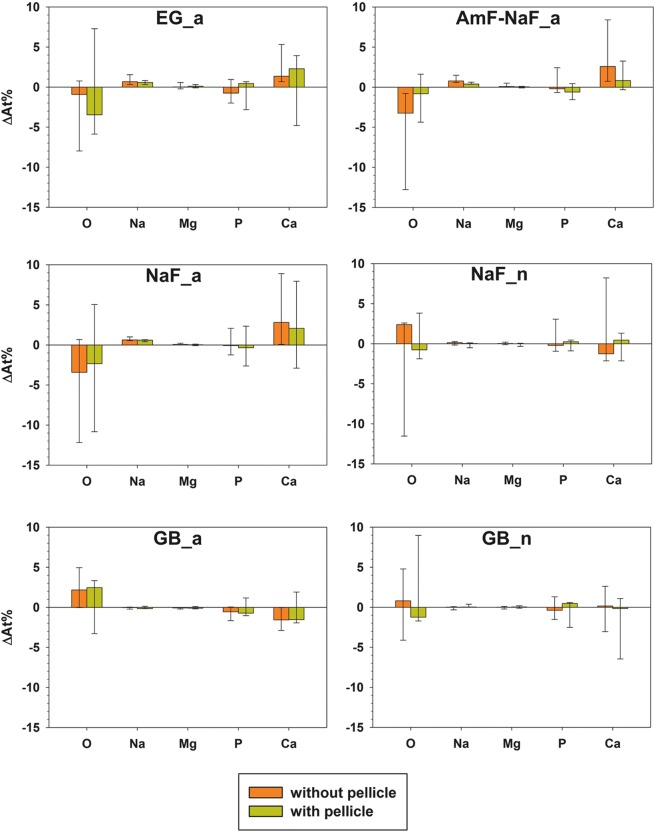
Difference (∆At%) between specimens with gel (minuend) and corresponding control specimens (subtrahend) of all elements after exclusion of F (Median and 25–75% percentiles).

Beyond fluorine, application of gel had an influence only on At%O and At%Ca. EG_a, AmF-NaF_a and NaF_a showed an increase in At%Ca, GB_a showed a decrease in At%Ca. NaF_n and GB_n did not show changes in At%Ca (Fig. 3).

**Figure 4:**
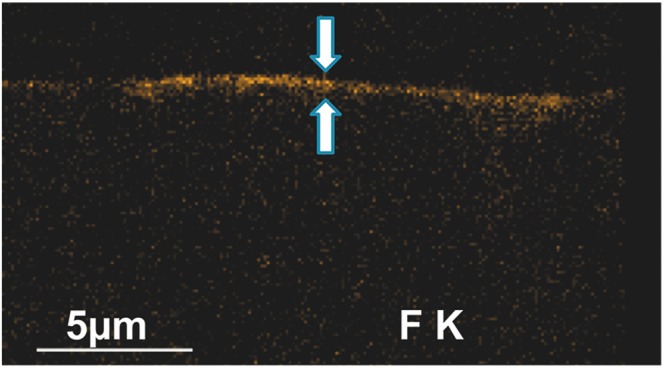
EDX F-Mapping of a cross-sectioned specimen from EG_a with pellicle shows characteristic layer of CaF_2_-precipitates (orange) with thickness of 400–610 nm (arrows).

The simulation of the ionization bulb generated by primary electrons during EDX-analysis shows a penetration depth for enamel and CaF_2_ in the order of 800 nm (data not shown). Figure 4 shows typical CaF_2_-precipitation in cross-sectional F-Mapping using EDX. Measured thickness of CaF_2_-precipitation (median, 25–75% percentile) of exemplary specimens in all groups following treatment with acidic fluoride gels regardless of the presence of salivary pellicle was 330 nm (280–400).

## Discussion

The ROI (x400 magnification) for EDX was chosen to be an area free of debris, microscratches or other artefacts due to material or processing conditions. It can be assumed that fluorine originally present in the outer layers of teeth, was removed by polishing procedures to obtain a surface devoid of inter-individual differences among teeth. At%C, At%Si and At%Cl were excluded during measurements to take into account artefacts due to storage in chloramine, polishing with silica suspension and sputtering with carbon.

**Figure 5:**
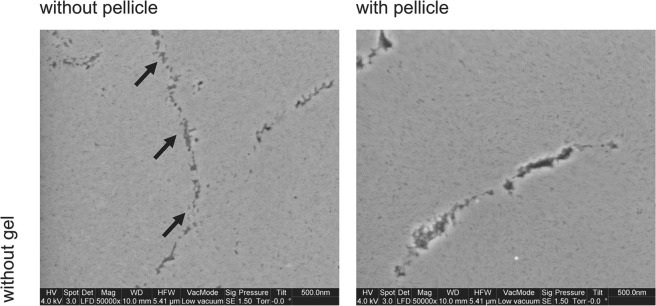
Typical images of control specimens (no gel application) without and with pellicle. Images show no superficial complexes in both specimens without gel but the granular structure of enamel crystals. The prism boundaries are indicated by black arrows (x50,000 original magnification, 5.41 µm horizontal field width).

All control specimens without gel application in absence or presence of a pellicle revealed 0 At%F. This is a necessary condition to detect At%F in test specimens without performing a correction. Morphology of a control specimen without precipitation is shown in Fig. 5. Moreover, all specimens treated with fluoride-free gel base, GB_a and GB_n, also revealed At%F <0.2. In all specimens treated with acidic fluoride containing gels the At%F was significantly higher indicating EDX as a reliable method to examine superficial fluoride-precipitation. In earlier studies KOH was used in order to dissolve CaF_2_-like precipitates and a fluoride-sensitive electrode was used to determine fluoride content in solution^21,23^. With our method in contrast, superficial surfaces are measured. The ion-selective electrode can be avoided and in addition to information on fluoride concentration, an SEM image is provided revealing insight into presence and distribution of such CaF_2_-like precipitates. A recent study using EDX as method revealed At%F > 0 in controls but without polishing^24^. This could be explained by posteruptive fluorine incorporation into the superficial enamel during de- and remineralization cycles. This condition should be taken into account before interpreting semiquantitative values. The problem becomes evident if control samples reveal higher At%F compared to fluoride treated surfaces. This occurred in the Hjortsjö-study for TiF_4_ and SnF_2_. The authors explained this with different mechanisms of these fluoride compounds.

All control specimens without and with pellicle revealed At%F-values between 0 At%F and 0.2 At%F. Therefore, it can be assumed that values above 0.2 At%F might be reliable to characterize superficial precipitations in this semiquantitative experimental setup. The established FSE (fluoride-sensitive electrode) method has a quantitative detection limit, but numerical values can not be compared with our study due to the fact that our data are semiquantitative^21,23^. With FSE, superficial fluorides must be dissolved with 1 M KOH solution for KOH-soluble fluoride content. Apart from that, structurally bound fluoride can be determined by using FSE an acidic solution of enamel powder gained by manually grinding or acid-dissolved enamel layers^20,22^.

Subsequently it can be assumed that CaF_2_-like precipitates equivalent to KOH-soluble fluoride and outer layers of the tooth with potential structurally bound fluoride are also included in one single EDX measurement.

With EDX, a direct detection of superficial compounds which can easily be attributed to specific locations upon the surface is possible, while using the FSE exact quantitative data can be generated.

Coefficients of variability of 13.7% for the three areas at maximum in groups with relevant fluoride deposition confirm EDX as a reliable method. One might conclude that a single measurement of At%F per specimen would be sufficient for further studies using this experimental setup. Fluorine content was expressed in relative terms to the other selected elements. Therefore, all ordinal scaled values have to be regarded semiquantitatively for every specimen. In this study O, Na, Mg, P and Ca were the most abundant elements which were included into the calculation of the At% in addition to fluorine.

In another study measuring percental elemental content by means of EDX, a maximum of 8.6% for fluorine was found. Varnishes with different amounts of TiF_4_ and NaF were applied in that study and Ca, P, F and Ti were included for calculation of elemental percentage^25^. Although in our study more elements were included, 12.3–15.3 At%F indicates a higher fluoride precipitation in our experimental setup. Simulation of the ionization bulb and morphologic SEM imaging of precipitates indicate that with a single measurement using EDX one may detect F^−^ from superficial precipitates and from outer enamel layers. Thus, structurally bound fluoride in outer enamel layers is also included.

It has been shown before that there is an intimate correlation between lower pH-values (pH < 7) of fluoride agents and amount of CaF_2_-like precipitates on enamel surfaces^12,26^.

However, not every fluoride agent seems to work primarily via pH-dependent precipitation of CaF_2_-like material. Fluoride peaks below untreated controls (0.5%) could be detected for acidic solutions with SnF_2_ (pH-value = 2.88) and TiF_4_ (pH-value = 1.58) in contrast to NaF and HF^24^. SnF_2_ led to precipitation of Sn, TiF_4_ led to precipitation of Ti.

In accordance to the results of Petzold and Hjortsjö *et al*., we measured fluorine peaks only for fluoride containing test materials with pH-value of 4.75. No precipitation was found for neutral NaF in our study. Higher Ca/P-ratios following enamel treatment with all acidic fluoride containing gels in the present study are indicative for CaF_2_-precipitation. From calculations of ∆At% it can be concluded that dissolved Ca-ions due to acidic pH can be bound to the surface in terms of superficial CaF_2_-precipitation.

**Figure 6:**
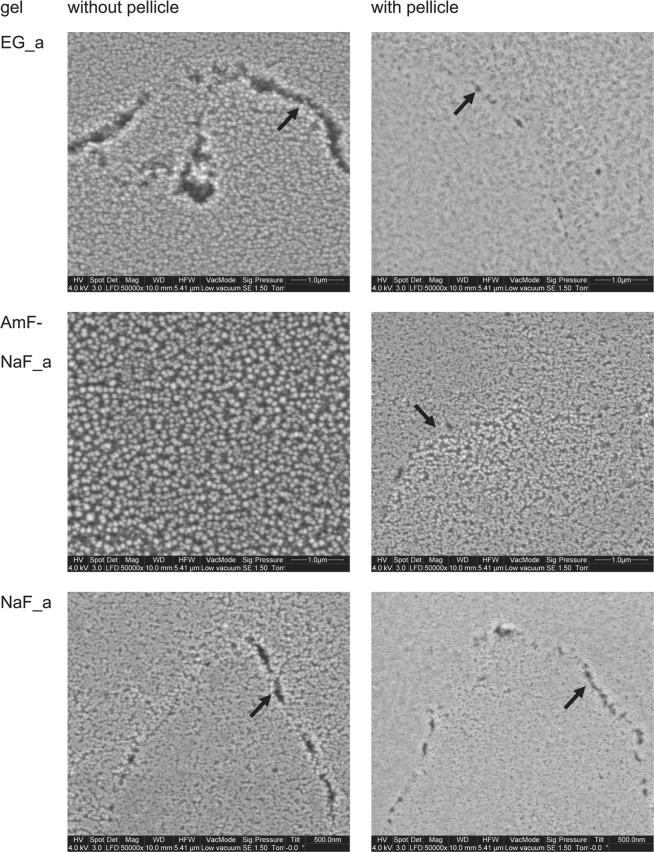
Characteristic morphological images of enamel surfaces after treatment with acidic fluoride gel; without pellicle (left column), with pellicle (right column); Globular CaF_2_-like structures with varying size and density are visible. Prism boundaries (black arrows) are visible in most cases (x50,000 original magnification, 5.41 µm horizontal field width).

**Figure 7:**
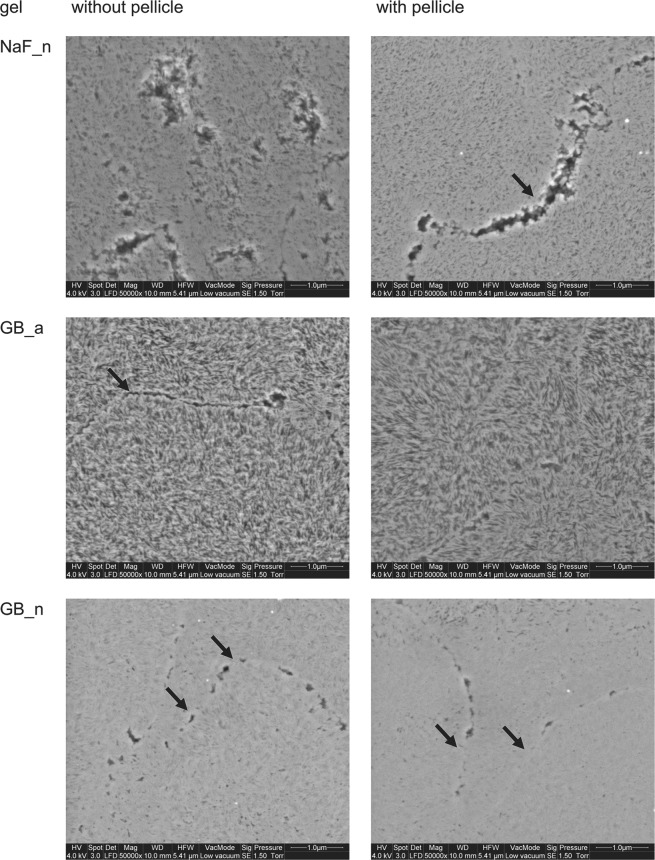
Characteristic morphological images of enamel surfaces after treatment with NaF_n, GB_a and GB_n without pellicle (left column) and with pellicle (right column); enamel crystals and prism boundaries (black arrows) are visible on all specimen. Application of the acidic gel base (GB_a) further exposed the crystals (x50,000 original magnification, 5.41 µm horizontal field width).

Several *in vitro* studies compared different exposure periods of acidulated phosphate fluoride. No significant differences between 1 and 4 minutes exposure were found regardless of whether varnishes or gels were used^26–28^. In this study, distinct precipitations formed during a 60 seconds exposure period (Fig. 6**)**. As exemplarily shown in Fig. 6, AmF-NaF_a resulted in larger globular precipitates than EG_a and NaF_a without pellicle. With pellicle, more indistinct but still dense precipitates showed for EG_a, AmF-NaF_a and NaF_a. A coarse enamel surface and etching patterns without precipitates were characteristic for gel bases GB_a and GB_n (Fig. 7). NaF_n exposure also showed enamel structures such as prism borders, but also less distinct superficial depositions in contrast with acidic gels (Fig. 7). The depicted images were acquired in Low Vacuum mode, which offered an evaluation of the native specimen immediately after specimen preparation without drying and sputtering. Although there is a loss of resolution in Low Vacuum mode due to water vapour in the SEM chamber, less artefacts of enamel surfaces are expected in this mode.

Besides exposure period and pH, the cation bound to fluorine in an ionic compound is an important factor for effectiveness of fluoride agents. Another *in vitro* study compared different TiF_4_-varnishes and NaF-varnishes using EDX depicting Ti, F, Ca and P. EDX revealed higher content of fluorine after treatment with TiF_4_ (up to 8.6%) as compared to NaF (up to 1.4%) in general. This can be explained by pH-value of 5 in all NaF-varnishes and pH-value of 1 in all TiF_4_ -varnishes^25^. Using the fluoride-sensitive electrode, higher values for KOH-soluble fluoride were detected only on bovine enamel. In contrast to TiF_4_-varnishes with their low pH-value of 1, the test materials used in the present study can be delegated to patients for home use^25^. Different experimental fluoride solutions containing NaF, HF and TiF_4_ in concentrations less than 1,000 ppm and SnF_2_ with 17,400 ppm revealed *in vitro* a maximum of 13.69 wt%F for 0.2% HF. Results are little consistent here as higher concentration of HF did not lead to more precipitation. Only HF and acidic NaF reached wt%F above 1^24^. The results of Hjortsjö *et al*. are in accordance with our study and the study of Petzold^12^ in terms of CaF_2_-precipitation through acidic NaF. Hjortsjö *et al*. observed no considerable fluoride-precipitation after treatment with TiF_4_ in contrast to Comar *et al*.

Fluorine peaks were detected using either NaF or combinations of NaF and AmF in the present study. It may be concluded that pH-value plays a more important role for fluoride-precipitation than specific cation content does. This was confirmed by lower Ca/P-ratio for neutral NaF as compared to NaF_a, AmF-NaF_a and EG_a. In our study, we examined gels with 12,500 ppm and 0 ppm exclusively. Using lower amounts of fluoride, e.g. 1,450 ppm as used in dentrifrices, might lead to less precipitation of CaF_2_ and lower At%F, possibly below the detection limit of our method, subsequently.

In a clinical context, adhesion of substances occurs to the pellicle at first, and after that they can possibly penetrate the pellicle^29^. Some other studies using EDX did not include saliva preconditioning^12,24^. In contrast to Comar *et al*.^25^, our approach was to establish a pellicle before application of test materials to mimic the clinical situation. The obtained pellicle significantly lowered At%F for NaF_a. This can be explained either by a barrier function of the pellicle or by an increase of pH-value due to the pellicle on the enamel interface. Although Error Rates Method revealed that general decrease of At%F with pellicle was not significant, values measured *in vitro* in absence of a pellicle might be misleadingly high.

## Methods, Test Materials and Controls

### Specimen preparation

30 caries-free third human molars, extracted for orthodontic reasons, were stored in 0.5% chloramine solution directly after extraction for a maximum of 3 months. Every crown was hand-sectioned into four equal sized specimens in bucco-oral and mesio-distal direction using a diamond disc (Horico Dental, Germany) under copious water cooling. Roots and pulpal tissue were removed. One central enamel region of each specimen was flattened and polished stepwise up to FEPA 4,000 with a water-cooled bench grinding machine (Metaserv Motopol 8, Buehler, Germany) with 3.6–4.0 rcf (200 rpm) for 80 seconds per step. Subsequently, every specimen was gently hand polished (MasterTex Vlies, Buehler; MasterMet 2, 0.02 µm silica suspension, Buehler) for 1 minute. Immediately after cleaning in an ultrasonic bath (Bransonic 221, Branson, USA) in demineralized water for 10 minutes the specimens were subjected to further treatments. In between the steps of the experimental procedure, the specimens were stored in demineralized water.

### Storage in saliva

**Figure 8:**
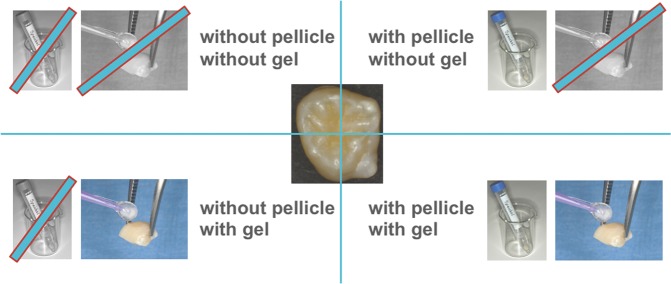
Treatment allocation of the four specimens of every tooth.

Saliva was collected from two healthy human donors after mechanical stimulation by chewing paraffin pellets (Ivoclar Vivadent, Liechtenstein), pooled and sterile-filtered (0.2 µm)^30,31^. Every tooth’s four specimens were treated as summarized in Fig. 8. Two out of the four specimens were deposited in saliva for 120 minutes to obtain a salivary pellicle and afterwards rinsed with demineralized water for 30 seconds. The remaining two specimens were stored in demineralized water during the same period of time.

### Gels and application

The compositions of the gels are shown in Table 3. The actual pH-value was measured in 10% solutions in aqua bidest just before application using a pH-meter (Inolab pH 7110, WTW GmbH, Germany). A marketed fluoride gel (EG_a, Elmex Geleé, CP GABA, USA) with acidic pH comprising NaF and amine fluoride (AmF) was taken as reference for comparison with five experimental formulations. The five experimental formulations were based on a hydroxyethyl cellulose gel. One experimental formulation (AmF-NaF_a) contained the same amount of amine fluoride (AmF) and sodium fluoride (NaF) and had the same pH as EG_a.

**Table 3:** Group allocation, gels, fluoride compound and pH-values; *10% solution in aqua bidest; AmF = amine fluoride, NaF = sodium fluoride; **a = acidic pH; ***n = neutral pH.

Group	Gel	Fluoride compound	Fluoride concentration [ppm F^−^]	aimed pH	measured pH * [median (Min-Max)]
EG_a **	Elmex Geleé (CP GABA, USA)	AmF, NaF	12,500	4.75	5.07 (4.95–5.1)
AmF-NaF_a	Experimental fluoride formulation	AmF, NaF	12,500	4.75	5.12 (5.04–5.27)
NaF_a	Experimental fluoride formulation	NaF	12,500	4.75	4.53 (4.52–4.61)
NaF_n ***	Experimental fluoride formulation	NaF	12,500	7.0	6.62 (6.35–6.64)
GB_a	Gel base	—	0	4.75	3.41 (3.38–3.58)
GB_n	Gel base	—	0	7.0	6.07 (5.78–6.31)

The second experimental gel (NaF_a) contained the same fluoride concentration and acidic pH as EG_a and AmF-NaF_a, but only NaF, not AmF. The third (NaF_n) was identical to NaF_a, but had a neutral pH. The other two experimental gels did not contain fluoride, but the gel base only at acidic (GB_a) and neutral (GB_n) pH. Five teeth (n = 5) per gel were used.

One enamel specimen each without and with pellicle was covered with the same gel. After carefully drying with paper wipes (KimTech Science Precision Wipes, Kimberly-Clark, UK) the gels were gently applied on the polished enamel surface with a saturated applicator tip (Flocked Applicator Tips, Dentsply International, USA) and left for 60 seconds. The specimens were rinsed with demineralized water using a squirt bottle for 30 seconds and immediately prepared for scanning electron microscopic inspection.

The other two specimens of each tooth were not treated with a gel. They were used as controls for measuring the basal intrinsic fluorine content of the enamel without or with pellicle.

### SEM visualisation

After the described application the enamel specimens were mounted onto aluminum stubs (Baltic Präparation, Germany) using acrylic resin (Palavit M, Heraeus, Germany) enriched with graphite powder (Baltic Präparation, Germany) without drying or contaminating the treated enamel surfaces. SEM-pictures (FEI Quanta 400 FEG, FEI Europe B.V., Netherlands) were taken in Low Vacuum mode (Secondary electron mode, Large Field Detector, 1.5 Torr, accelerating voltage 4 kV, WD = 10 mm, aperture Ø 30 µm, image resolution 2048 × 1768 pixels, range of magnification x6,000–50,000).

### EDX

**Figure 9:**
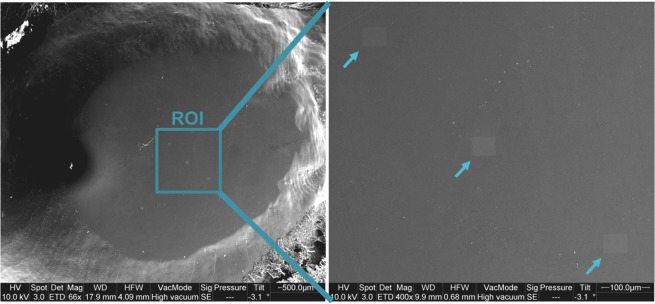
Area selection. Left section shows definition of ROI within the center of the polished enamel surface (x66 original magnification, 4.09 mm horizontal field width). Right section shows imprints of the SEM beam (arrows) after EDX determination (x400 original magnification, 0.68 mm horizontal field width).

After documentation in the SEM, the specimens were dried for five days in an exsiccator, using activated silica gel (Silica-Gel with indicator/Orange-Gel, 1–3 mm, Merck, Germany). All specimens were carbon coated (carbon doublefibers, Baltic Präparation, Germany; WD = 30 mm, vacuum <5 × 10^−2^ mbar, 23–30 nm layer thickness). Subsequently, the elemental composition of the surface was examined by semiquantitative energy-dispersive X-ray analysis (EDX; EDAX micro analysis system, Octane Plus Silicon Drift Detector, “TEAM Enhanced” v. 4.3). Atomic percent (At%) of the elements O, F, Na, Mg, P and Ca were measured in this study. At%F was the target parameter of the experiment. C, Cl and Si were not included in the analysis. The EDX measurements were performed using High Vacuum mode (HV; 10^−4^–10^−6^ Torr, accelerating voltage 10 kV, WD = 10/12 mm, aperture Ø 50 µm, measurement time 200 live seconds, image resolution 1024 × 800 pixels, magnification x6,000). On each specimen a region of interest (ROI) was defined located centrally in the polished surface of the respective specimen. Within the ROI, three fields (49.7 × 38.8 µm at magnification x6,000) in one diagonal line were defined for elemental analysis (Fig. 9).

Two-dimensional simulation of the ionization bulb generated by primary electrons was performed using CASINO software version 2.51 (10 keV, beam radius 10 nm, take off 35°, tilt of specimen 0°)^32–34^ Parameters for sound enamel were the chemical formula of hydroxy apatite Ca_10_(PO_4_)_6_(OH)_2_ with a density of 3.021 g/cm^3^ ^35^. CaF_2_ was simulated as well with a density of commercially available CaF_2_ of 3.18 g/cm^3^ ^36^.

### Data analysis

For EDX-data, nonparametric statistical analyses were used to analyze At%F (SPSS version 25.0, SPSS Inc., USA). The median of the three measured areas per ROI was used as the representative value of every specimen. Coefficients of variability of the At%F were calculated from the three scanned areas of every specimen. Group-Medians, 25%- and 75%-percentiles from specimens’ representative values were determined for each gel. Mann-Whitney-U-Test was used to test for statistically significant differences between groups. The level of significance was set to α = 0.05. For evaluation of pellicle’s influence in general, the level of significance α was adjusted to α*(k) = 1-(1-α)^1/k^ with the Error Rates Method (k = number of paired tests performed).

∆At% without and with pellicle between specimens with gel application minus control specimens were calculated after exclusion of F, C, Cl and Si for all remaining elements to determine influences of gels.

### Ethical approval

The use of extracted human teeth was approved by the Ethics Committee (Faculty of Medicine, University of Regensburg, Regensburg, Germany; Nr. 19-1327-101) after obtaining informed consent form each patient. All experiments were performed in accordance with relevant guidelines and regulations.

## Conclusions

In conclusion, within the limitations of the present *in vitro* study, EDX appeared as a reliable and relatively fast method to semiquantitatively measure superficial CaF_2_-precipitates in terms of At%F. The pellicle modulates but does not prevent formation of CaF_2_-like structures on enamel surfaces. The increased formation of CaF_2_-like precipitates at a lower pH-value was confirmed and supported by changes in Ca/P-ratio and ∆At% of O, Na, Mg, P and Ca. Highest accumulation of At%F was found after treatment with acidic fluoride formulations on surfaces without pellicle.

## Data Availability

All relevant data are within the paper files.
